# Formal comment on: Piscine reovirus: Genomic and molecular phylogenetic analysis from farmed and wild salmonids collected on the Canada/US Pacific Coast

**DOI:** 10.1371/journal.pone.0188690

**Published:** 2017-11-30

**Authors:** Molly J. T. Kibenge, Yingwei Wang, Alexandra Morton, Richard Routledge, Frederick S. B. Kibenge

**Affiliations:** 1 Department of Pathology and Microbiology, Atlantic Veterinary College, University of Prince Edward Island, Charlottetown, Prince Edward Island, Canada; 2 School of Mathematical and Computational Sciences, University of Prince Edward Island, Charlottetown, Prince Edward Island, Canada; 3 Raincoast Research Society, Sointula, British Columbia, Canada; 4 Department of Statistics and Actuarial Science, Simon Fraser University, Burnaby, British Columbia, Canada; National Cheng Kung University, TAIWAN

## Introduction

This formal comment is in response to Siah *et al*. [1], Piscine Reovirus: Genomic and Molecular Phylogenetic Analysis from Farmed and Wild Salmonids Collected on the Canada/US Pacific Coast, with a subsequent correction (Siah *et al*. 2016 [2]). Although a correction for this paper was published on Oct. 12, 2016, (Siah *et al*. 2016 [2]), there continues to be inadequate supporting evidence for the primary conclusion that PRV genetic sequences are temporally and spatially homogeneous in salmonid species across the northeastern Pacific region.

The evidence in this paper warrants thorough consideration. Piscine orthoreovirus (PRV) causes acute infection of the red blood cells in salmon (Finstad *et al*. 2014 [3]; Haatveit *et al*. 2017 [4]). It is the causative agent of the emerging farm salmon disease Heart and Skeletal Muscle Inflammation (HSMI) (Wessel *et al*. 2017 [5]) with clinical symptoms which can include lethargy, anemia, anorexia and mortality (Kongtorp *et al*. 2004 [6]). Palacios *et al*. [7] expressed concerns about the transfer of PRV from farmed to wild fish due to its contagious nature. PRV is now considered ubiquitous in farmed Atlantic salmon (Haatveit *et al*. 2017 [4]) and has an estimated 80% prevalence rate among BC farmed salmon (Kibenge *et al*. 2013 [8]). HSMI has recently been diagnosed in British Columbia (BC), Canada (Di Cicco *et al*. 2017 [9]). Hence, release of PRV from salmon farms into Pacific salmon habitat is a significant management concern in the eastern Pacific Ocean.

In the correction, Siah *et al*. [2] acknowledge that the conclusion that PRV has not been recently introduced to BC was overstated. However their supporting evidence that "… salmonids from western North America Pacific waters carried PRV RNA sequences for at least 13 years with little genetic differentiation among sequence types in selected samples spanning 2001 to 2014" remains insufficient.

Their conclusion appears to be highly dependent on six unique sequences of PRV segment S1, detected by Siah *et al*. [1]:

KR478642: collected in May 2001
KR478643: collected in Aug. 2001
KR478644: collected in Aug. 2001
KR347078: collected in Aug. 2001
KR347079: collected in Aug. 2001
KR347080: collected in Mar. 2005

These six Siah *et al*. [1] sequences collected in 2001 and 2005 (submitted to GenBank April—May 2015) predate those collected by Kibenge *et al*. [10,11] by seven years and are nearly identical to the isolates Siah *et al*. [1] collected in 2013 and 2014. Thus, these six PRV isolates appear to be highly resistant to mutation over a 13-year interval 2001–2014, which is atypical for RNA viruses, generally known to exhibit a high mutation rate (Chao *et al*. [12]). Drake and Holland [13] estimate the genomic mutation rate (*U*) to be between 1 and 0.1 for most RNA viruses, where *U* is *G* x *u*, *G* is the genome size in nucleotides, and *u* is the per-nucleotide mutation rate.

### Weight of evidence for longer-term PRV presence in BC

Siah et al. [1] cite detection of PRV in a wild Steelhead trout (*O*. *mykiss*) collected in 1977 in support of longer-term PRV presence in BC. This result is cited from Marty *et al*. [14], who provided no S1 segment sequence information to verify the PRV strain identity as per the sequence groupings reported by both Kibenge *et al*. [8] and Garseth *et al*. [15]. The recent discovery of the widespread occurrence of PRV-2 across the North Pacific (Takano *et al*. 2016 [16]) raises the question: Was the 1977 steelhead infected with PRV-2 or PRV? In absence of S1 sequencing this uncertainty cannot be resolved.

Furthermore, this result could not be replicated by a second laboratory (Purcell and Thompson 2014 [17]) and therefore warrants qualification as a non-repeatable result and a suspect positive lacking sufficient robustness to provide evidence critical to the temporal presence of PRV in BC.

### Phylogenetic comparative analysis

**Fig 1:**
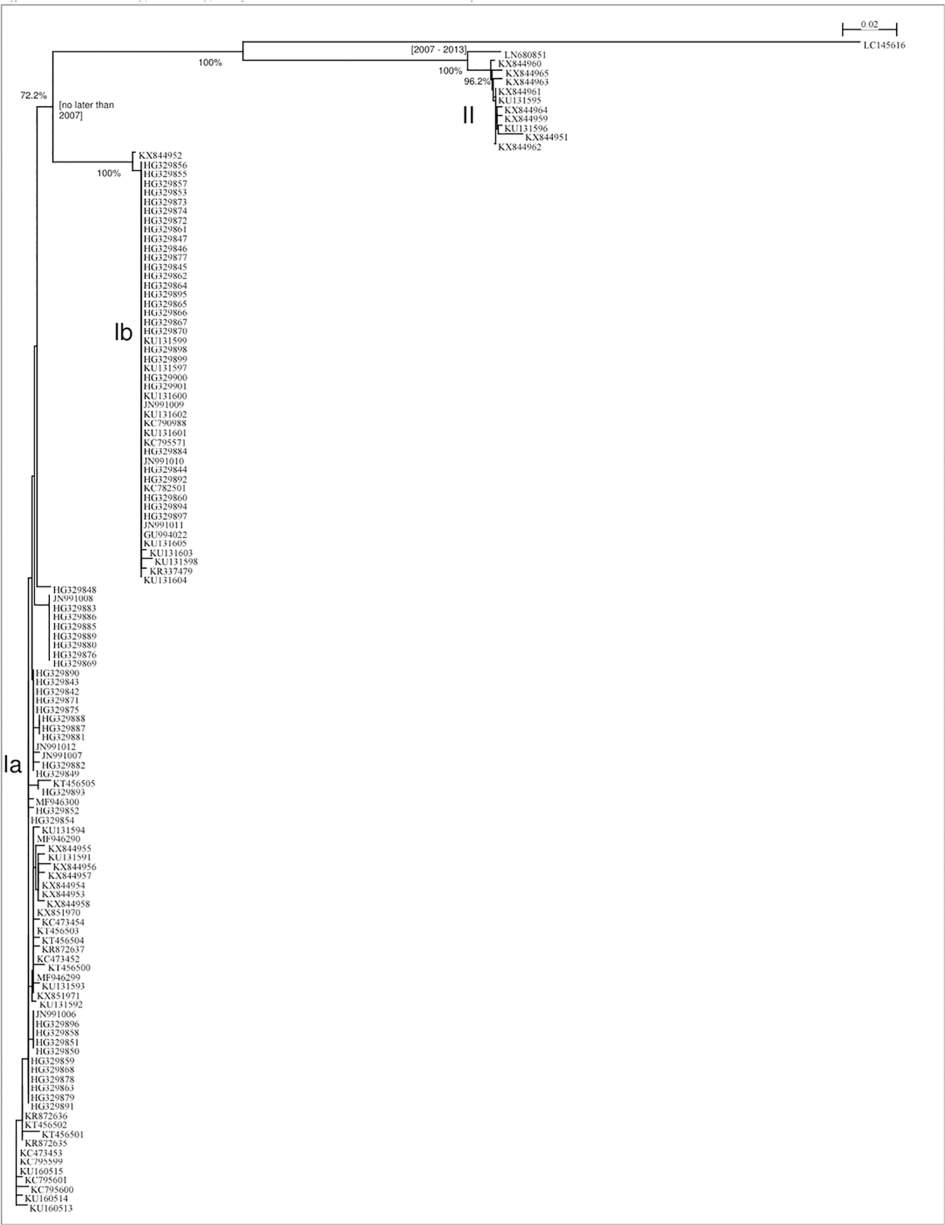
Phylogenetic tree for sequences of piscine orthoreovirus (PRV) segment S1 listed in S1 Table. All 127 available robust isolates are included in this tree. The phylogenetic tree was constructed using the neighbor-joining method and Tamura-Nei genetic distances (Saitou and Nei 1987 [18]). Bootstrapping was performed 1,000 times. Bootstrap supports of topology of 70% or higher are shown at the nodes. The PRV grouping of Genotype I sub-genotypes Ia, Ib, and Genotype II are indicated. The mutation direction was determined by an outgroup sequence.

To illustrate our interpretation of the phylogenetic analysis of PRV isolates, we constructed a phylogenetic tree (Fig 1), of the 127 sequences described in S1 Table. The mutation direction was determined by an outgroup sequence (GenBank Accession No. AF004856). After the root of the tree was determined, the outgroup sequence was removed so that the details of the tree could be shown.

Fig 1 demonstrates that all PRV isolates can be classified into Genotypes I and II, with Genotype I further divided into sub-genotype Ia and sub-genotype Ib. Among these sub-genotypes, all Canadian isolates exist in sub-genotype Ia; this evidence provides information that PRV in BC-Canada is closely related to PRV found in Norway.

We estimated the divergence time between the genotypes and sub-genotypes (Ia, Ib and II) based on the collection time of each isolate described in S1 Table. Basic rules of logic were used in the estimation, such as the divergence time must not be later than the collection time of any isolate in all branches.

We estimated that the divergence time between sub-genotype Ia and sub-genotype Ib was 2007 or earlier. Because all sequences in S1 Table were collected in 2007 or later, we are not able to estimate a more accurate divergence time. We also estimated that the divergence time between Genotype II and the rest of the isolates was in the range of 2007 to 2013. The PRV-2 sequence GenBank Accession No. LC145616, from Japan (Takano *et al*. 2016 [16]), is quite different from all other isolates and it may constitute a second sub-genotype of Genotype II or a completely new genotype (Genotype III); but because we cannot find other evidence, we consider this sequence an outlier at the present time.

### Sampling inadequacies

The description of the temporal and spatial distribution of the samples reported on by Siah *et al*. [1] is inadequate to support the conclusion that PRV is endemic to BC. The reader cannot ascertain the degree of independence between sampled fish. For example, there are two groups of samples that generated PRV sequences from locations labeled “Hatchery (British Columbia, Canada)”. One group was sampled in October 2013, and another eight samples were obtained in November 2013. Were these fish from the same, related (i.e. shared personnel and broodstock) or different hatcheries? Fish sampled one month apart from the same hatchery would presumably have an increased probability of being infected with the same strain of PRV and cannot be interpreted as independent samples.

Furthermore, all but eight of the partial sequences reported by Siah *et al*. [1] in their Table 2 were obtained in 2013 and 2014. Of the eight other sequences, two were from 2012, one from 2005, and the remaining five were from 2001 (four of which were reportedly sampled on the same day on one or more farms within DFO Statistical Area 18; an area with no reported marine salmon farms, suggesting these were from a hatchery). Thus, it appears that there were only three temporally separate sampling events for the time period 2001–2012. This sparse temporal coverage does not provide sufficiently extensive evidence to support a conclusion of long-term PRV genetic heterogeneity in BC.

There was also inadequate spatial and host-species coverage. Siah *et al*. [1], reported that over half (43/71) of the fish that produced partial PRV sequence information were farmed Atlantic salmon (Siah *et al*. 2015 [1] Table 2). Only two wild fish were sampled north of central BC (two Coho Salmon, *O*. *kisutch*, from the Copper River in Alaska), and only six Chinook Salmon, *O*. *tshawytscha*, were sampled (four from southern BC, and two from further south in the Columbia River, Washington State). No other Pacific salmon or trout species were included. Hence, the host species and spatial coverage of PRV sequencing presented by Siah *et al*. [1] are very sparse. Thus Siah *et al*. [1] do not provide sufficient evidence to draw reliable inferences either on the temporal stability or geographic homogeneity of PRV throughout the coastal eastern Pacific Ocean. Considerable variation over time or space could easily have been bypassed.

Furthermore, farm restocking methods could potentially account for at least some of the homogeneity in the Siah *et al*. [1] samples. Although BC farm salmon broodstock sourcing and the distribution of Atlantic salmon from specific hatcheries is not public information in BC, presumably farm salmon from the same hatchery could be transferred into farms hundreds of kilometers apart that are sited throughout wild eastern Pacific salmon migratory corridors. Repeat introduction of the same PRV variant across years and regions may be occurring from Atlantic salmon hatcheries that share broodstock and/or eggs. Thus, the appearance of genetic stability of PRV in migratory wild salmon could be the result of exposure to farm salmon from the same hatchery.

### HSMI in BC

Siah *et al*. [1] state, citing Kibenge *et al*. [8] and Marty *et al*. [14], “PRV is known to occur in a wide variety of salmon species on the Pacific Coast of North America, *a region where HSMI has never been reported*.” (Emphasis added.) However, Kibenge *et al*. [8] do cite lesions identified as diagnostic of HSMI in BC farmed Atlantic salmon beginning in 2008. Furthermore, subsequent to both these publications, HSMI has been confirmed in BC (Di Cicco *et al*. 2017 [9]).

## Conclusion

We conclude that the longer-term presence of PRV in BC prior to 2001 has not been adequately described and that the evidence that the virus was introduced from Norway is more robust than the hypothesis that PRV is endemic to the eastern Pacific Ocean.

## Supporting information

S1 Table Piscine orthoreovirus segment S1 nucleotide sequences analyzed in this study. (DOC)
