# Supplementary material for: Formal comment on: Piscine reovirus: Genomic and molecular phylogenetic analysis from farmed and wild salmonids collected on the Canada/US Pacific Coast
Source: PLoS One. 2017 Nov 30;12(11):e0188690. doi: 10.1371/journal.pone.0188690 (PMC5708765; doi:10.1371/journal.pone.0188690)
Supplement: S1 Table — (DOC) [file pone.0188690.s001.doc]

**S1 Table:** Piscine orthoreovirus segment S1 nucleotide sequences analyzed in this study

| **PRV isolate ID** | **Country** | **GenBank Accession number** | **Reference** | **Year** |
| --- | --- | --- | --- | --- |
| VT02292012-163 | Canada | KC473452 | Kibenge et *al.* [1] | 2012 |
| VT06062012-358 | Canada | KC473453 | Kibenge et *al.* [1] | 2012 |
| VT06202012-371 | Canada | KC473454 | Kibenge et *al.* [1] | 2012 |
| VT02292012-167 | Canada | KC795599 | Kibenge et *al.* [1] | 2012 |
| VT03202012-196 | Canada | KC795600 | Kibenge et *al.* [1] | 2012 |
| VT03202012-209 | Canada | KC795601 | Kibenge et *al.* [1] | 2012 |
| VT09122012-755 | Canada | KT456500 | Kibenge and Kibenge [2] | 2011 |
| VT09192013-402 | Canada | KT456501 | Kibenge and Kibenge [2] | 2013 |
| VT09192013-408 | Canada | KT456502 | Kibenge and Kibenge [2] | 2013 |
| VT01212014-03 | Canada | KT456503 | Kibenge and Kibenge [2] | 2014 |
| VT01212014-04 | Canada | KT456504 | Kibenge and Kibenge [2] | 2014 |
| VT01212014-09 | Iceland | KT456505 | Kibenge and Kibenge [2] | 2014 |
| VT01292015-09 | Canada | KU160513 | Kibenge and Kibenge [2] | 2015 |
| VT07222015-106 | Canada | KU160514 | Kibenge and Kibenge [2] | 2015 |
| VT07222015-107 | Canada | KU160515 | Kibenge and Kibenge [2] | 2015 |
| VT02162017-59 | Norway | MF946299 | Kibenge et *al.* [3] | 2017 |
| VT03022017-69 | Canada | MF946300 | Kibenge et *al.* [3] | 2017 |
| VT10092015-122 | Canada | MF946290 | Kibenge et *al.* [3] | 2017 |
| BCinoc3 | Canada | KR872635 | Garver *et al.* [4] | 2012 |
| BCinoc12_13 | Canada | KR872636 | Garver *et al.* [4] | 2013 |
| BC361_14 | Canada | KR872637 | Garver *et al.* [4] | 2014 |
| B5690 | Canada | KX851970 | DiCicco et al. [5] | 2013 |
| B7274 | Canada | KX851971 | DiCicco et al. [5] | 2013 |
| 5433-S3 | Norway | JN991006 | Løvoll *et al.* [6] | 2012 |
| 1921-S3 | Norway | JN991007 | Løvoll *et al.* [6] | 2012 |
| 9326-S3 | Norway | JN991008 | Løvoll *et al.* [6] | 2012 |
| 3817-S3 | Norway | JN991012 | Løvoll *et al.* [6] | 2012 |
| 35 Bjoreio | Norway | HG329842 | Garseth *et al.* [7] | 2009 |
| 45 Eira | Norway | HG329843 | Garseth *et al.* [7] | 2009 |
| 131 Gaula | Norway | HG329848 | Garseth *et al.* [7] | 2009 |
| 182 Hestdal | Norway | HG329849 | Garseth *et al.* [7] | 2009 |
| 187 Hestdal | Norway | HG329850 | Garseth *et al.* [7] | 2009 |
| 190 Hestdal | Norway | HG329851 | Garseth *et al.* [7] | 2009 |
| 211 Mandal | Norway | HG329852 | Garseth *et al.* [7] | 2009 |
| 246 Mandal | Norway | HG329854 | Garseth *et al.* [7] | 2009 |
| 284 Stjordal | Norway | HG329858 | Garseth *et al.* [7] | 2009 |
| 307 Stjordal | Norway | HG329859 | Garseth *et al.* [7] | 2009 |
| 407 Vosso | Norway | HG329863 | Garseth *et al.* [7] | 2009 |
| 445 Alta | Norway | HG329868 | Garseth *et al.* [7] | 2008 |
| 470 Drevja | Norway | HG329869 | Garseth *et al.* [7] | 2008 |
| 517 Eira | Norway | HG329871 | Garseth *et al.* [7] | 2008 |
| 629 Jolstra | Norway | HG329875 | Garseth *et al.* [7] | 2008 |
| 708 Nausta | Norway | HG329876 | Garseth *et al.* [7] | 2008 |
| 842 Vikja | Norway | HG329878 | Garseth *et al.* [7] | 2008 |
| 851 Vikja | Norway | HG329879 | Garseth *et al.* [7] | 2008 |
| 866 Vikja | Norway | HG329880 | Garseth *et al.* [7] | 2008 |
| 907 Vosso | Norway | HG329881 | Garseth *et al.* [7] | 2008 |
| 909 Vosso | Norway | HG329882 | Garseth *et al.* [7] | 2008 |
| 931 Alta | Norway | HG329883 | Garseth *et al.* [7] | 2007 |
| 985 Ekso | Norway | HG329885 | Garseth *et al.* [7] | 2007 |
| 987 Ekso | Norway | HG329886 | Garseth *et al.* [7] | 2007 |
| 989 Ekso | Norway | HG329887 | Garseth *et al.* [7] | 2007 |
| 993 Ekso | Norway | HG329888 | Garseth *et al.* [7] | 2007 |
| 1039 Laerdal | Norway | HG329889 | Garseth *et al.* [7] | 2007 |
| 1062 Mandal | Norway | HG329890 | Garseth *et al.* [7] | 2007 |
| 1137 Stjordal | Norway | HG329891 | Garseth *et al.* [7] | 2007 |
| 1195 Aaroy | Norway | HG329893 | Garseth *et al.* [7] | 2007 |
| 1343 Moelv | Norway | HG329896 | Garseth *et al.* [7] | 2008 |
| VT12202013_CGA_2013_4 | Chile | KU131591 | Godoy *et al.* [8] | 2013 |
| VT12202013_CGA_2013_1 | Chile | KU131592 | Godoy *et al.* [8] | 2013 |
| VT12202013_CGA_2013_2 | Chile | KU131593 | Godoy *et al.* [8] | 2013 |
| 2015_CGA_2015_B | Chile | KU131594 | Godoy *et al.* [8] | 2015 |
| VT12202013_CGA_2013_3 | Chile | KU131595 | Godoy *et al.* [8] | 2013 |
| VT12202013_CGA_2013_5 | Chile | KU131596 | Godoy *et al.* [8] | 2013 |
| 61 Eira | Norway | HG329844 | Garseth *et al.* [7] | 2009 |
| 81 Etne | Norway | HG329845 | Garseth *et al.* [7] | 2009 |
| 90 Etne | Norway | HG329846 | Garseth *et al.* [7] | 2009 |
| 93 Etne | Norway | HG329847 | Garseth *et al.* [7] | 2009 |
| 220 Mandal | Norway | HG329853 | Garseth *et al.* [7] | 2009 |
| 261 Nidelv | Norway | HG329855 | Garseth *et al.* [7] | 2009 |
| 273 Skibotn | Norway | HG329856 | Garseth *et al.* [7] | 2009 |
| 283 Skjomen | Norway | HG329857 | Garseth *et al.* [7] | 2009 |
| 318 Storelva Holt | Norway | HG329860 | Garseth *et al.* [7] | 2009 |
| 338 Surna | Norway | HG329861 | Garseth *et al.* [7] | 2009 |
| 350 Surna | Norway | HG329862 | Garseth *et al.* [7] | 2009 |
| 411 Vosso | Norway | HG329864 | Garseth *et al.* [7] | 2009 |
| 412 Vosso | Norway | HG329865 | Garseth *et al.* [7] | 2009 |
| 414 Vosso | Norway | HG329866 | Garseth *et al.* [7] | 2009 |
| 438 Alta | Norway | HG329867 | Garseth *et al.* [7] | 2008 |
| 491 Eira | Norway | HG329870 | Garseth *et al.* [7] | 2008 |
| 522 Ekso | Norway | HG329872 | Garseth *et al.* [7] | 2008 |
| 555 Fusta | Norway | HG329873 | Garseth *et al.* [7] | 2008 |
| 565 Gaula | Norway | HG329874 | Garseth *et al.* [7] | 2008 |
| 818 Surna | Norway | HG329877 | Garseth *et al.* [7] | 2008 |
| 982 Eira | Norway | HG329884 | Garseth *et al.* [7] | 2007 |
| 1160 Surna | Norway | HG329892 | Garseth *et al.* [7] | 2007 |
| 1261 Halsan | Norway | HG329894 | Garseth *et al.* [7] | 2009 |
| 1309 Eidsdal | Norway | HG329895 | Garseth *et al.* [7] | 2008 |
| 1361 Moelv | Norway | HG329897 | Garseth *et al.* [7] | 2008 |
| 1459 Etne | Norway | HG329898 | Garseth *et al.* [7] | 2010 |
| 1462 Etne | Norway | HG329899 | Garseth *et al.* [7] | 2010 |
| 1463 Etne | Norway | HG329900 | Garseth *et al.* [7] | 2010 |
| 1469 Etne | Norway | HG329901 | Garseth *et al.* [7] | 2010 |
| 7243-S3 | Norway | JN991009 | Løvoll *et al.* [6] | 2012 |
| 7030-S3 | Norway | JN991010 | Løvoll *et al.* [6] | 2012 |
| 8286-S3 | Norway | JN991011 | Løvoll *et al.* [6] | 2012 |
| Salmo/GP-2010/NOR | Norway | GU994022 | Palacios *et al.* [9] | 2010 |
| 050607 | Norway | KR337479 | Haatveit *et al.* [10] | 2007 |
| CGA337 | Chile | KC782501 | Kibenge *et al.* [1] | 2012 |
| CGA8857 | Chile | KC790988 | Kibenge *et al.* [1] | 2012 |
| CGA280-05 | Chile | KC795571 | Kibenge *et al.* [1] | 2012 |
| 2013_CGA_2013_A | Chile | KU131597 | Godoy *et al.* [8] | 2013 |
| 2013_CGA_2013_C | Chile | KU131598 | Godoy *et al.* [8] | 2013 |
| VT02182014_CGA_2013_8 | Chile | KU131599 | Godoy *et al.* [8] | 2013 |
| VT02182014_CGA_2013_9 | Chile | KU131600 | Godoy *et al.* [8] | 2013 |
| VT02182014_CGA_2013_10 | Chile | KU131601 | Godoy *et al.* [8] | 2013 |
| VT02182014_CGA_2013_6 | Chile | KU131602 | Godoy *et al.* [8] | 2013 |
| VT02182014_CGA_2013_7 | Chile | KU131603 | Godoy *et al.* [8] | 2013 |
| 2015_CGA_2015_A | Chile | KU131604 | Godoy *et al.* [8] | 2015 |
| 2015_CGA_2015_C | Chile | KU131605 | Godoy *et al.* [8] | 2015 |
| F445-2013 | Norway | LN680851 | Olsen *et al.* [11] | 2013 |
| C10/P1.1 | Chile | KX844965 | Cartagena *et al.* [12] | 2014 |
| C10/P1.2 | Chile | KX844964 | Cartagena *et al.* [12] | 2014 |
| C10/P2.1 | Chile | KX844963 | Cartagena *et al.* [12] | 2014 |
| C10/P2.2 | Chile | KX844962 | Cartagena *et al.* [12] | 2014 |
| C10/P3.1 | Chile | KX844961 | Cartagena *et al.* [12] | 2014 |
| C10/P3.2 | Chile | KX844960 | Cartagena *et al.* [12] | 2014 |
| C10/P4.2 | Chile | KX844959 | Cartagena *et al.* [12] | 2014 |
| V/P1.1 | Chile | KX844958 | Cartagena *et al.* [12] | 2014 |
| V/P1.2 | Chile | KX844957 | Cartagena *et al.* [12] | 2014 |
| V/P2.1 | Chile | KX844956 | Cartagena *et al.* [12] | 2014 |
| V/P2.2 | Chile | KX844955 | Cartagena *et al.* [12] | 2014 |
| V/P3.1 | Chile | KX844954 | Cartagena *et al.* [12] | 2014 |
| V/P3.2 | Chile | KX844953 | Cartagena *et al.* [12] | 2014 |
| IM/P5.2 | Chile | KX844952 | Cartagena *et al.* [12] | 2015 |
| C10/P4.1 | Chile | KX844951 | Cartagena *et al.* [12] | 2014 |
| PRV-2 | Japan | LC145616 | Takano *et al.* [13] | 2012 |

**References**

1. Kibenge MJT, Iwamoto T, Wang Y, Morton A, Godoy, MG, Kibenge FSB. Whole-genome analysis of piscine reovirus (PRV) shows PRV represents a new genus in family Reoviridae and its genome segment S1 sequences group it into two separate sub-genotypes. Virol J 2013: 10:230.
2. Kibenge FSB, Kibenge MJ. 2015: Piscine reovirus segment S1 sequences from fish samples from British Columbia-Canada. Direct submissions to GenBank database.
3. Kibenge FSB, Kibenge MJ, Morton A. 2017: Piscine reovirus segment S1 sequences from fish samples from British Columbia-Canada. Direct submissions to GenBank database.
4. Garver KA, Marty GD, Cockburn SN, Richard J, Hawley L, Müller A, Thompson RL, et al. Piscine reovirus, but not jaundice syndrome, was transmissible to Chinook salmon, Oncorhynchus tshawytscha (Walbaum), Sockeye salmon, Oncorhynchus nerka (Walbaum), and Atlantic salmon, Salmo salar (L.). J Fish Dis 2016: 39:117-128. doi: 10.1111/jfd.12329
5. Di Cicco E, Ferguson HW, Schulze AD, Kaukinen KH, Li S, Vanderstichel R, et al. Heart and skeletal muscle inflammation (HSMI) disease diagnosed on a British Columbia salmon farm through a longitudinal farm study. 2017; PLoS ONE 2017: 12(2): e0171471. https://doi.org/10.1371/journal.pone.0171471
6. Løvoll M, Alarcón M, Jensen BB, Taksdal T, Kristoffersen, AB, Tengs T. Quantification of piscine reovirus (PRV) at different stages of Atlantic salmon Salmo salar production. Dis Aquat Organ 2012: 99:7-12
7. Garseth, ÅH, Ekrem T, Biering E. Phylogenetic evidence of long distance dispersal and transmission of piscine reovirus (PRV) between farmed and wild Atlantic salmon. PLoS ONE 2013;8:e82202.
8. Godoy MG, Kibenge MJT, Wang Y, Suarez R, Leiva C, Vallejos F, Kibenge FSB. First description of clinical presentation of piscine orthoreovirus (PRV) infections in salmonid aquaculture in Chile and identification of a second genotype (Genotype II) of PRV. Virol J. 2016: 13:98
9. Palacios, G, Lovoll, M, Tengs, T, Hornig, M, Hutchison, S, Hui, J, et al. Heart and skeletal muscle inflammation of farmed salmon is associated with infection with a novel reovirus. PLoS One 2010: 5(7):e11487. doi: 10.1371/journal.pone.0011487 PMID: 2392291
10. Haatveit HM, Nyman IB, Markussen T, Wessel O, Dahle MK, Rimstad E. 2015: The non-structural protein muNS of piscine reovirus (PRV) forms viral factory-like structures. GenBank Accession Numbers KR337473-KR337482 represent sequences from the 10 segments of Piscine reovirus isolate 050607. Direct submissions to GenBank database.
11. Olsen AB, Hjortaas M, Tengs T, Hellberg H, Johansen R. First Description of a new disease in rainbow trout (Oncorhynchus mykiss (Walbaum)) similar to heart and skeletal muscle inflammation (HSMI) and detection of a gene sequence related to piscine orthoreovirus (PRV). PLoS ONE 2015: 10:e0131638.
12. Cartagena J, Tambley C, Sandino AM, Spencer E, Tello M. 2016: Detection of a virus related to Piscine orthoreovirus (PRV) in *O. mykiss* affected by idiopathic syndrome of rainbow trout (ISRT). GenBank Accession Numbers KX844958-KX844965. Direct submissions to GenBank database.
13. Takano T, Nawata A, Sakai T, Matsuyama,T, Ito T, Kurita J, et al. Full-Genome Sequencing and Confirmation of the Causative Agent of Erythrocytic Inclusion Body Syndrome in Coho Salmon Identifies a New Type of Piscine Orthoreovirus. PLoS ONE 2016: 11(10): e0165424.doi:10.1371/journal.pone.0165424
